# Mechanical Signaling in the Sensitive Plant *Mimosa pudica* L.

**DOI:** 10.3390/plants9050587

**Published:** 2020-05-04

**Authors:** Takuma Hagihara, Masatsugu Toyota

**Affiliations:** 1 Department of Biochemistry and Molecular Biology, Saitama University, Saitama 338-8570, Japan; t.hagihara.517@ms.saitama-u.ac.jp; 2 Department of Botany, University of Wisconsin, Madison, WI 53706, USA

**Keywords:** *Mimosa pudica* L., mechanical stimulation, turgor pressure, action potential, long-distance signaling

## Abstract

As sessile organisms, plants do not possess the nerves and muscles that facilitate movement in most animals. However, several plant species can move quickly in response to various stimuli (e.g., touch). One such plant species, *Mimosa pudica* L., possesses the motor organ pulvinus at the junction of the leaflet-rachilla, rachilla-petiole, and petiole-stem, and upon mechanical stimulation, this organ immediately closes the leaflets and moves the petiole. Previous electrophysiological studies have demonstrated that a long-distance and rapid electrical signal propagates through *M. pudica* in response to mechanical stimulation. Furthermore, the spatial and temporal patterns of the action potential in the pulvinar motor cells were found to be closely correlated with rapid movements. In this review, we summarize findings from past research and discuss the mechanisms underlying long-distance signal transduction in *M. pudica*. We also propose a model in which the action potential, followed by water flux (i.e., a loss of turgor pressure) in the pulvinar motor cells is a critical step to enable rapid movement.

## 1. Introduction

Unlike animals, plants are often regarded as immotile organisms, as they do not normally make movements that can be easily observed by humans. However, in nature, plants can slowly displace their bodies in response to various stimuli. Such types of slow movements (minutes to hours) are widely seen in plants as they adapt to various environmental pressures. For instance, many plant organs can sense changes in the sunlight direction and bend their bodies towards it accordingly, a phenomenon called phototropism [1]. Furthermore, a variety of plants such as leguminous species are known to open and fold their leaves (nyctinastic movement) in response to the diurnal light/temperature cycle [2]. These slow movements have been studied since the days of Charles and Francis Darwin [3].

On the other hand, some plant species can move rapidly (within seconds) in response to external stimuli; for example, in response to mechanical stimulation, the sensitive leguminous plant *Mimosa pudica* quickly folds its leaves. Many studies have documented the propagation of stimulus information induced by mechanical stimuli (e.g., [4,5,6,7,8]). This rapid movement and long-distance signaling have been the focus of numerous significant research, yet there are still many gaps remaining in our knowledge of the mechanisms underlying rapid plant movements and stimuli-induced signal transduction. In this review, we will discuss the current consensus on mechanical signaling for rapid movement and long-distance signal propagation in *M. pudica*.

## 2. Rapid Movements

**Figure 1:**
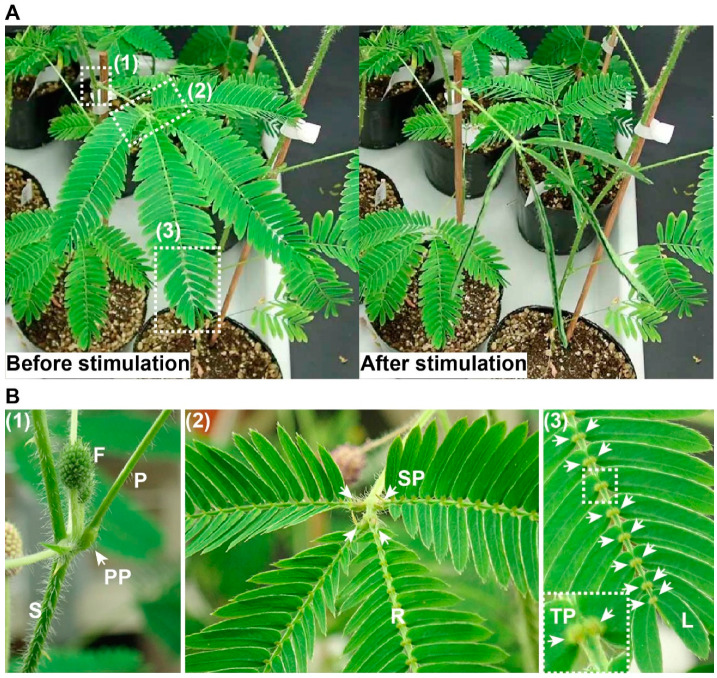
Rapid movement characteristics in *M. pudica*. (**A**) The leaf of *M. pudica*, shown expanded before mechanical stimulation (left panel) and folded after the stimulation (touched by hand, right panel). (**B**) The motor organs pulvini. (1) Primary pulvinus (PP, white arrow). S, stem; F, floral bud; P, petiole. (2) Secondary pulvini (SP, white arrows). R, rachilla (the central axis of the pinna). (3) Tertiary pulvini (TP, white arrows). The inset shows a magnified image of pairs of tertiary pulvini enclosed by the dashed line in (3). L, leaflet. The numbers in each panel correspond to those shown in (**A**).

When a leaf of *M. pudica* is mechanically stimulated (e.g., touched by hand), it drops and leaflets are folded upward (Figure 1A). Such rapid movements are only possible due to the existence of the pulvini, found at the base of leaflet, rachilla, and petiole (Figure 1B). Of the three types of pulvini found in *M. pudica*, the primary and tertiary pulvini are more sensitive to mechanical stimuli than the secondary pulvinus. Early literature reported that movement was still generated in *M. pudica* even when the upper half of the primary pulvinus (the flexor side) was removed (described in [9]), suggesting that the lower half (the extensor side) plays an essential role in the generation of rapid movements. Asprey and Palmer [9] also tested this hypothesis using a different experimental condition wherein they horizontally placed a leaf to disregard leaf weight on the primary pulvinus from which either the extensor or flexor half was removed and found that both the extensor and flexor halves of the primary pulvinus were necessary for movement. Subsequently, researchers observed a redistribution of water from the extensor to the flexor half in the primary pulvinus after movement [10]. These findings led to the view that sudden turgor loss on the extensor side of the pulvinus caused by water redistribution was the driving force behind rapid movements in plants.

Generally, turgor-driven movements (e.g., nyctinastic leaf movement) are triggered by the release of electrolytes such as K^+^ and Cl^−^ from extensor motor cells [11]. Supporting this idea, Toriyama [12] reported that K^+^ migrated from the intracellular to the extracellular space of the motor cells after movement, suggesting that water efflux osmotically driven by K^+^ migration results in the turgor loss of the pulvinus of *M. pudica*. This phenomenon was confirmed by Allen [13] using radioactive ^42^K^+^. It was later demonstrated that extracellular (apoplastic) Cl^−^ concentration also increases during movement [14]. Extensor cells release more Cl^−^ than the flexor cells and Cl^−^ migration from the pulvinar cells is initiated immediately, before, or almost simultaneously with rapid pulvinar bending [14]. Several reagents affecting K^+^ or Cl^−^ flux across the plasma membrane inhibit movement, supporting the idea that the migration of these ions is a critical precursor event for turgor loss and movement in the pulvinus [15]. Although ion channels on the motor cells’ plasma membrane in *M. pudica* have not yet been identified, the outwardly rectified K^+^ current, which is activated by membrane depolarization, has been electrophysiologically described [16]. In addition to K^+^ and Cl^−^, it has also been reported that photoassimilates (such as sucrose) may be associated with rapid movement in *M. pudica*. Fromm and Eschrich [17] observed that ^14^C-labeled photoassimilates, which were restricted to the phloem of the pulvini, were found in the apoplastic regions of other tissues after stimulation (e.g., in the extensor motor cells in the pulvini).

Water flux across the plasma membrane/tonoplast and the corresponding leaf movements are linked to a specific membrane protein: the water channel aquaporin [18]. Aquaporins are therefore thought to contribute to the rapid movement in *M. pudica* [19]. Fleurat-Lessard et al. [19] found that the density of aquaporins on the tonoplasts of mature pulvini was higher than that of less sensitive younger pulvini, which is, in turn, correlated with their motility. The plasma membrane aquaporins have also been identified from *M. pudica* seedlings and their activity has been confirmed by expressing them in *Xenopus laevis* oocytes [20], supporting the idea that water flux through aquaporins is involved in the rapid movement.

Toriyama [21] reported that stimulation induced the migration of unknown colloidal substances from the central vacuole to the extracellular space in *M. pudica*, suggesting these were diffused from the motor cells simultaneously with K^+^ migration [12]. Furthermore, Samejima and Sibaoka [14] proposed that the sap efflux from the central vacuole decreases the volume of motor cells, resulting in rapid pulvinar bending. These studies suggest that the effusion of vacuolar substances from motor cells may directly regulate their volume.

The above phenomena driving movement led to the need to identify the “trigger” signal required for initiating movement, which researchers suspect to be the action potential. Early attempts to measure electrical signals were primarily made using galvanometers. This meant that while the electrical activities of *M. pudica* could not be precisely traced, the electrical changes in the pulvinus could be observed [22]. As electrophysiological measurement techniques were developed further, they allowed researchers to obtain more accurate measurements and monitor the action potentials generated at primary pulvini [14,23,24]. These action potentials were generated before rapid movements [14,25] and Cl^−^ efflux from pulvinar cells [14]. Although rapid movement and action potential generation are related in space and time, the mechanisms underlying the perception of mechanical stimuli are yet to be determined. Recently, a putative mechanoreceptor cell on the tertiary pulvinus has been identified using electrophysiological and anatomical techniques [26]. The mechanoreceptor cells, which are bright red in color, seem to be derived from stomatal subsidiary cells and generate a receptor potential in response to mechanical stimulation [26]. These cells also have direct connections to excitable motor cells via plasmodesmata in tertiary pulvini [26]. Thus, these cells should contain mechanoreceptors to convert mechanical stimuli into electrical signals. As applying a mechanical force changes the structural properties of the cell surface (e.g., plasma membrane and cell wall), stretch-activated or receptor-type ion channels may be suitable for mechanoreceptors to transduce external stimuli into electrical signals [26]. The membrane stretch that occurs upon mechanical stimulation is known to directly activate stretch-activated channels in plants [27]. Damage-associated molecular patterns (DAMPs) are molecular elicitors released upon wounding, such as peptides, ATP, and oligogalacturonides derived from the plant cell wall pectin and activate various plant defense responses [28]. Although mechanoreceptors and DAMPs have not yet been identified in *M. pudica*, the physical force exerted in the plasma membrane/cell wall or molecular elicitors could trigger the initial electrical signals and thus movement.

To investigate the electrical properties of the pulvinus in *M. pudica,* Volkov et al. [29] electrically stimulated the primary pulvinus using a charged capacitor. They then constructed a model of an equivalent electrical circuit in the pulvinus based on the electrically anisotropic character of the pulvinus. If the circuit is activated by stimulation, voltage-dependent channels could be opened to drive rapid plant movement [29].

**Figure 2:**
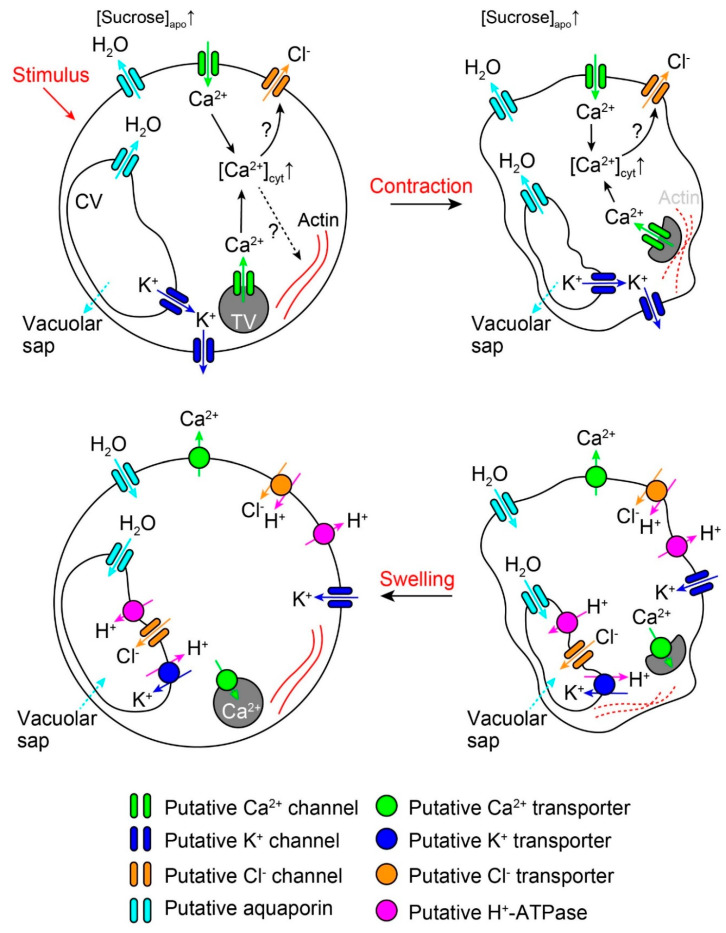
The turgor loss model for rapid movement in *M. pudica*. Extensor motor cells release electrolytes into the apoplastic region in response to a mechanical stimulus. Subsequently, water is osmotically released into the extracellular space. The electrolytes are transferred through voltage-dependent K^+^ and Cl^−^ channels, which are activated by the action potential. Ca^2+^ influx via the plasma membrane and Ca^2+^ efflux from the tannin vacuole result in an increase in [Ca^2+^]_cyt,_ which activate a Ca^2+^-dependent Cl^−^-channel to depolarize the plasma membrane. The increase in [Ca^2+^]_cyt_ might also facilitate rapid movements via actin filament depolymerization. Alternatively, the central vacuole may release its sap through an unknown mechanism in response to a mechanical stimulus (dashed blue arrows, top panels). In the recovery phase, the effused substances and water are taken back into the cell to restore the turgor (bottom panels). H^+^-ATPase provides the energy required for the active uptake of effused substances. K^+^ is transferred into the cells via an inwardly rectified K^+^ channel. Ca^2+^ migrates to tannin vacuoles and the apoplastic region via a Ca^2+^ pump. CV, central vacuole; TV, tannin vacuole.

Using microscopic and pharmacological techniques, the potential relationship between the rapid movement of *M. pudica* and Ca^2+^ has also been tested. The tannin vacuole is a specialized vacuole containing the polyphenol tannin, which coexists with the central vacuole in the motor cell (Figure 2). Previous research has shown that tannin vacuoles contain Ca^2+^ before movement, but hardly any afterward, suggesting that this structure acts as a Ca^2+^ store [30]. Furthermore, when tertiary pulvini were treated with the conventional Ca^2+^ channel blocker La^3+^, or the divalent cation chelator EDTA, they gradually lost their ability to fold [31]. The effect of EDTA was reversed following additional treatment with Ca^2+^ to the pulvini [31]. Moreover, various Ca^2+^ channel inhibitors (e.g., nifedipine) have been found to retard the movement of the primary pulvinus, while the Ca^2+^ channel agonist, Bay-K 8644, increases the velocity of the pulvinar movement [32,33]. When alterations in the Ca^2+^ concentration in the motor cell protoplasts were monitored with a Ca^2+^ fluorescent dye (Fura-2), EGTA and ruthenium red inhibited both the increases in cytosolic Ca^2+^ concentration ([Ca^2+^]_cyt_) and contractile movements [33]. These findings suggest that Ca^2+^ plays an essential role in triggering rapid movements in *M. pudica,* possibly mediated by the increase in [Ca^2+^]_cyt_. This latter mechanism has not yet been elucidated, but the knowledge of Ca^2+^ dynamics in nyctinastic movements may be useful to explain the role of [Ca^2+^]_cyt_ in the rapid movement of *M. pudica*. Red light irradiation, which causes phytochrome-mediated nyctinastic leaf closure, generated transient [Ca^2+^]_cyt_ increases in motor cells of *Robinia pseudoacacia* and this Ca^2+^ could be partly derived from the cell wall [34]. Therefore, Ca^2+^ influx into a motor cell might directly depolarize the plasma membrane, resulting in the activation of voltage-dependent K^+^ and/or Cl^−^ channels [34], or a [Ca^2+^]_cyt_ increase may activate Ca^2+^-dependent Cl^−^ channels, followed by turgor loss during nyctinastic slow movement (and possibly the rapid movement) of *M. pudica*.

The reorganization of actin filaments could be associated with rapid plant movements. Fluorescence labeling and confocal microscopy studies showed that actins formed filaments in motor cells before mechanical stimulation, but that these same filaments were fragmented after stimulation [33,35]. When primary pulvini were treated with reagents to inhibit the polymerization or de-polymerization of actin filaments, pulvinar movements were successfully inhibited, suggesting that the reorganization of the actin filaments was critical for movement [33,35]. A protein from the Gelsolin/Fragmin family, which modulates the degree of actin polymerization, has been previously identified from *M. pudica* [36]. Intriguingly, this isolated protein severed actin filaments in a Ca^2+^-dependent manner in vitro [36], suggesting that the increase of [Ca^2+^]_cyt_ in motor cells might regulate both ion fluxes and actin depolymerization during movement. Meanwhile, phalloidin, an inhibitor for actin depolymerization, retarded the increase in [Ca^2+^]_cyt_ in the motor cell protoplast, the actin filament might also regulate changes in [Ca^2+^]_cyt_ [33]. On top of their fragmentation during movement, the level of phosphorylation of the actin filaments is likely to be important for movement: actin filaments are phosphorylated before movement and dephosphorylated after [37]. Inhibitors for tyrosine phosphatase decreased pulvinar bending [35,37] while serine/threonine phosphatase inhibitors failed to retard the movement, suggesting that selective phosphorylation of tyrosine residue in actin controls movement [35].

In nyctinastic leaf movements, it is thought that organic compounds modulate the slow movements [38]. The chemicals consisting of “leaf-opening factors” and “leaf-closing factors” were isolated and identified in several nyctinastic plant species, including in *M. pudica*, which have different molecular structures for each plant species [38]. Interestingly, these chemicals only function in plants from which they were extracted [38]. When these factors were labeled with fluorescence indicators, they were found to be mainly located in the extensor motor cells of the pulvinus, suggesting that specific receptor proteins for these factors exist in these cells [38]. Nyctinastic movements are generally easily distinguished from rapid movements, but the possible additional contribution of these factors to rapid movements cannot be excluded.

Although the aforementioned studies were largely based on experiments examining the primary pulvinus, the findings can clarify the mechanism of rapid bending initiated in the tertiary pulvinus. This led to the creation of a turgor loss model driving pulvinar movements (Figure 2). In this model, electrolytes (e.g., K^+^ and Cl^−^) and photoassimilates (e.g., sucrose) migrate to the apoplast upon mechanical stimulation and water is simultaneously released into the extracellular space, leading to turgor loss in the motor cells. Mechanical stimulation generates receptor potential in the mechanoreceptor cells and the following action potential in the motor cells. The action potential may trigger Ca^2+^ influx into the motor cells, which in turn directly triggers membrane depolarization. Depolarization-activated ion channels then induce an efflux of electrolytes; or a [Ca^2+^]_cyt_ increase activates Ca^2+^-dependent Cl^−^ channels to depolarize the plasma membrane and voltage-dependent K^+^ channels transfer K^+^ into the extracellular region. Alternatively, the central vacuole releases its sap with small molecules (e.g., colloidal substance), reducing the motor cell volume. In the recovery phase, the effused substances and ions are actively transported into the motor cell and water is then osmotically pulled back into the cell. The ion uptake appears to be coupled to the H^+^ gradient produced by the activity of H^+^-ATPases [19,39]. It is also possible that motor cells absorb apoplastic K^+^ through inward-rectifier K^+^ channels, which are activated by H^+^ pump-driven plasma membrane hyperpolarization [11] or other mechanisms [40]. The central vacuole is an intracellular store of solutes and water, suggesting that the vacuole releases or accumulates ions using channels and transporters during motor cell shrinking or swelling, as with the stomatal movement [41]. Cytosolic Ca^2+^ could be taken back into a tannin vacuole and apoplast assisted by Ca^2+^ pump, as the Ca^2+^ pump inhibitors delay the recovery phase of movement [32]. Beside this osmotic water uptake, it is thought that the effused vacuolar sap is returned into the central vacuole and, therefore, the cell volume is restored.

## 3. Long-Distance Signaling

When *M. pudica* is stimulated, it generates a stimulus-induced signal propagating toward distant organs. Early studies examining this phenomenon monitored the pulvinar movement as a visible indicator of signal transmission [4,5]. Ricca (described in [4]) reported that stimuli could pass through the stem region separated by the water-filled tube in *M. spegazzinii* (a sensitive relative of *M. pudica*). As applying the stem extract solution to the cut end of the shoot generated signal propagation, he concluded that this conduction was a result of stimulating chemicals released from wounded tissues through water transported in the xylem vessels (described in [4]). This phenomenon was later confirmed in *M. pudica* by Snow [4]. Since then, several studies have been published which use electrophysiological methods to reveal a variety of long-distance signal propagations.

Houwink [6] conducted electrophysiological experiments, suggesting that *M. pudica* possesses three types of signals in mechanosensory transduction as follows: (1) an action potential, (2) a variation potential, and (3) a rapid unknown signal (quicker than the previous ones). The variation potential is an electrical signal triggered by wounding (e.g., flame and cut) [6]. Propagation of the variation potential is thought to be linked to a flow of stimulating chemicals in the xylem vessels upon stimulation [6]. Therefore, this signal could propagate from a wounded site to distant organs through the xylem and pulvinus, and is hence different from that of the action potential. Furthermore, the velocity of this signal is slower than that of the action potential [6]. The rapid unknown signal may be a non-electrical signal induced by cutting off organs such as a pinna [6]. The mechanism of this signal transduction is largely unknown, but this putative signal could move the primary pulvinus and petiole after pruning a pinna earlier than the arrival of the action and variation potentials [6]. In this review, however, we mainly focus on the action potential.

The action potential is the most commonly observed signal in *M. pudica* and has a steep spike-like waveform. Houwink [6] referred to the action potential as the “action current” and described it as “the action of living cells.” The action potential can be generated by both wounding and non-wounding stimuli (e.g., a chilled water drop) [6]. When pairs of recording electrodes are placed across the primary or secondary pulvini, the electrode placed on the stimulated side (before the pulvinus) can detect the action potential but not after the pulvinus after a non-wounding stimulus, suggesting that the action potential cannot propagate over the pulvini [6,7]. When an intracellular measurement method was introduced to detect the true action potential generated by a single cell, excitable cells in various tissues in *M. pudica* were examined. These studies reported that excitable cells in petioles were parenchyma cells in the protoxylem and parenchyma or companion cells in the phloem [42,43]. Using an aphid stylet as a passage to the sieve tube in the petiole, the transmission of the action potential was also confirmed in the sieve tube [44]. These vascular excitable cells should contribute to the propagation of the action potential in *M. pudica* [42,43,44]. Samejima and Sibaoka [24] attempted to understand the relationship between the generation of the action potential and the ion composition (ion strength) in the extracellular fluid in which the target tissues were immersed. They found that the amplitudes of the action potentials recorded from excitable protoxylem cells in the petioles were affected by external Cl^−^ concentration [24]. Subsequently, it was also reported that the amplitudes of the action potentials obtained from excitable cells in the petioles immersed into Ca^2+^-free extracellular fluid were smaller than those in the Ca^2+^-containing solution [43]. These findings suggest that Ca^2+^ and Cl^−^ both contribute to action potential generation in *M. pudica*, but that further analysis is required to obtain conclusive evidence.

As discussed, the action potential of *M. pudica* had been studied in detail and is widely thought to be one of the mechanisms of stimulus transmission. However, this hypothesis is not yet fully accepted. In a study where a part of the leaf is burned, changes in the leaf thickness of others, more distant leaves emerged almost immediately [8]. This result prompted the hypothesis that there is a “hydraulic signal” passing through the xylem, which may facilitate the dispersal of chemicals from the wounded tissues and cause the local electrical response (such as the action potential) in the excitable cells [8].

## 4. Conclusions and Perspectives

In this review, we conclude that electrical signals, in particular action potentials, are likely to be important for the rapid movement and long-distance signaling in *M. pudica*. While a large part of the underlying mechanisms is yet to be determined, the contribution of several ions and cytoskeleton playing a role in these events has been suggested. Most studies investigating mechanical signaling in *M. pudica* are old, and until now, research on this species has largely relied on microscopic, pharmacological, electrophysiological, and histochemical techniques or their combined methods. Generally, legumes are supposed to be difficult to transform [45] and there is no genomic information on *M. pudica*, unlike other plants (e.g., the model plant *Arabidopsis*). As such, genetic approaches have rarely been used for research on *M. pudica.* However, recently a stable transformation method using Agrobacterium was developed [46], enabling the use of genetic approaches in studies on *M. pudica*, as various intra and extracellular signals are visualized in real-time in *Arabidopsis* and *Nicotiana* species [47,48,49]. This methodological breakthrough is expected to open up many new avenues of research into mechanical signaling using *M. pudica*.
